# Resting-state Functional Connectivity within Frontoparietal Network in Schizophrenia Patients and Healthy Individuals with Better and Worse Executive Functions

**DOI:** 10.1192/j.eurpsy.2022.415

**Published:** 2022-09-01

**Authors:** Y. Panikratova, E. Abdullina, D. Tikhonov, V. Kaleda, I. Lebedeva

**Affiliations:** 1 Mental Health Research Center, Laboratory Of Neuroimaging And Multimodal Analysis, Moscow, Russian Federation; 2 Mental Health Research Center, Department Of Youth Psychiatry, Moscow, Russian Federation

**Keywords:** resting-state fMRI, schizophrénia, frontoparietal network, Executive functions

## Abstract

**Introduction:**

Patients with schizophrenia spectrum disorders (SP) demonstrate heterogeneity in executive functions (EF) associated with the quality of outcome. However, neurobiological mechanisms of this heterogeneity are understudied.

**Objectives:**

We aimed to identify features of resting-state functional connectivity (FC) within the frontoparietal network (FPN) that discriminate between SP and healthy individuals (HI) with better and worse EF.

**Methods:**

Twenty-five SP (mean age 20.8±3.23, illness duration 1.3±2.1 years, all males) and twenty-six HI (mean age 25.17±3.46, all males) underwent EF assessment (4 verbal fluency tests and a modified Stroop task) as well as resting-state fMRI (3T).

**Results:**

We used *k*-means clustering based on EF scores to divide all participants into groups with worse (15 SP, 6 HI) and better EF (10 SP, 20 HI). These groups differed in productivity of all verbal fluency tasks and performance time of the Stroop task. Differences between four subgroups (HI/SP with worse/better EF) were revealed in FC between the cingulate and lateral prefrontal cortex in the left hemisphere (ANCOVA, *p*-uncorrected<.005, *p*[FDR]<.05; Fig. 1). SP and HI within each group demonstrated a similar FC pattern. SP with poorer EF had increased FC, compared to HI with higher EF. HI with poorer EF demonstrated increased FC, compared to HI and SP with better EF.

**Figure:**
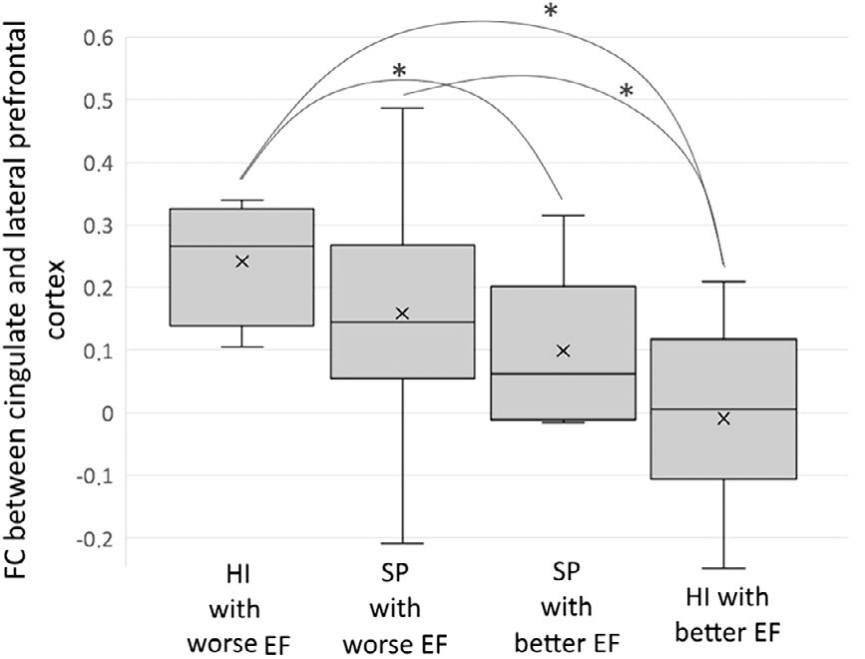

**Conclusions:**

FC within FPN may be one of the neurophysiological underpinnings of EF heterogeneity in SP as well as in HI. Further machine learning fMRI studies are needed to clarify whether FC within FPN is a prognostic marker in schizophrenia.

**Disclosure:**

The study was supported by RFBR Grant 20-013-00748.

